# Genomic and Phenotypic Characterization of Sandstorm-Derived Airborne *Bacillus* Strains Reveals Diverse Biosynthetic Potential

**DOI:** 10.3390/antibiotics15090863

**Published:** 2026-09-04

**Authors:** Khadijah M. Dashti, Leila Vali, Nawal Mohammed, Ali A. Dashti

**Affiliations:** 1 Department of Medical Laboratory Sciences, Faculty of Allied Health Sciences, Kuwait University, Kuwait City 46300, Kuwait; ali.dashti2@ku.edu.kw; 2 School of Education and Applied Science, Francis Close Hall Campus, University of Gloucestershire, Cheltenham GL50 2RH, UK; lvali@glos.ac.uk; 3 Hematology and Blood Banking Laboratory, Farwaniya Hospital, Farwaniya 81004, Kuwait; mnawal48@gmail.com

**Keywords:** *Bacillus*, sandstorm, secondary metabolites, antimicrobial resistance

## Abstract

**Background/Objectives**: *Bacillus* spp. are often isolated from airborne microbiota. Five *Bacillus* strains (AD12–16) isolated from dust during a sandstorm in Kuwait were characterized for their potential to produce antimicrobial compounds, biosurfactants and siderophores by genotypic and phenotypic approaches. **Methods**: Whole-genome sequencing was performed, and biosynthetic gene clusters (BGCs) were predicted using antiSMASH. Antimicrobial activities were determined by agar-well diffusion assays. Siderophore and biosurfactant production were determined by Arnow’s and drop-collapse assays, respectively. The active metabolites were separated, fractionated, and analyzed by minimum inhibitory concentration (MIC), minimum bactericidal concentration (MBC) and LC–MS/MS. **Results**: Genome mining identified many BGCs associated with known secondary metabolites such as bacillibactin and bacilysin, as well as a number of unassigned clusters. The isolates exhibited distinct antimicrobial profiles, with *Bacillus subtilis* AD16 displaying the highest antimicrobial activity against multidrug-resistant pathogens. Biosurfactant- and siderophore-associated activities varied among the isolates. Active fractions demonstrated bactericidal activity, while LC–MS/MS analysis revealed molecular features tentatively assigned to siderophore- and lipopeptide-associated metabolite classes based on accurate mass and MS/MS fragmentation patterns. **Conclusions**: The combination of unassigned BGCs, antimicrobial activity, and incompletely characterized molecular features highlights unexplored biosynthetic potential in sandstorm-derived *Bacillus*. Further purification and structural characterization are required to establish the identities and biological functions of the active metabolites.

## 1. Introduction

Antimicrobial resistance (AMR) is a global crisis that causes millions of deaths annually and severely affects current therapeutic plans in healthcare systems [1]. It is associated with five million deaths annually, and the number continues to rise as multidrug-resistant pathogens continue to spread [1]. The World Health Organization (WHO) recently reported the dissemination and spread of multidrug-resistant pathogens as a critical priority that needs to be addressed immediately [2]. With the increase in the spread of antibiotic-resistant pathogens, there is also a decline in the discovery of antibiotics [1]. One of the best-known sources of antimicrobial compounds is environmental microbes; however, many of them are still underexplored [3]. *Bacillus* species are one of the most prolific producers of antimicrobial secondary metabolites (SMs) [3]. These species are known producers of structurally and chemically diverse antimicrobial secondary metabolites, including polyketides such as bacillaene, lipopeptide antibiotics such as fengycins, surfactins, and polymyxins, as well as other prominent antimicrobial compounds such as bacitracin, gramicidin, and bacilysin [3]. Most of these antimicrobial compounds are encoded by biosynthetic gene clusters (BGCs), which are groups of genes that together code for the biosynthesis of secondary metabolites [4]. However, some of these BGCs are not expressed under standard laboratory conditions and therefore are termed silent or “cryptic” [4].

The Whole-Genome Sequence (WGS) of bacteria, including *Bacillus* species or any other environmental bacteria, can be used to predict silent biosynthetic pathways, some of which are involved in antimicrobial production, using genome mining tools such as antiSMASH, PRISM, and BAGEL [5]. Moreover, these predicted BGCs can be linked to experimental antimicrobial activity to fully integrate the genotype with the phenotype [6]. Most studies have investigated the antimicrobial potential of *Bacillus* species isolated from plants, marine, or soil habitats [7]. However, less attention has been given to airborne *Bacillus* species, especially those isolated during extreme sandstorm events [8]. Sandstorm-induced atmospheric changes commonly cause the transport and spread of dense aerosols that contain bacteria, fungi, and spores across other regions [8,9].

*Bacillus* species are considered frequent dominant members of the airborne microbiome due to their spore-forming potential [10]. Kuwait, which has a desert climate and is located in the northern Arabian Peninsula, frequently experiences dust storms with high particulate and microbial loads [8]. A recent environmental monitoring study in Kuwait has reported diverse airborne *Bacillus* species isolated from indoor hospital air and outdoor air during a sandstorm [8]. Although antimicrobial activity and secondary metabolite production are well established among *Bacillus* spp., the biosynthetic diversity and chemically unresolved antimicrobial metabolites of *Bacillus* strains recovered from sandstorm-associated airborne dust remain underexplored [7,8]. In particular, the occurrence of unassigned BGCs in sandstorm-derived *Bacillus* strains may indicate biosynthetic potential that warrants further genomic and metabolomic characterization.

Microorganisms exposed to harsh environmental conditions, including ultraviolet radiation, desiccation, nutrient limitations, and oxidative stress, can be affected in their ecological adaptation and biosynthetic potential [11]. Recent evidence from long-distance-transported dust microbial communities demonstrated that *Bacillus* spp. employ biofilm formation and niche adaptation strategies that facilitate persistence during atmospheric transport, highlighting the importance of multicellular organization and adaptive responses in dust-associated *Bacillus* populations [9]. Moreover, recent studies have shown that atmospheric microbiomes are transported during dust events and can harbor metabolically versatile microorganisms with diverse secondary metabolite BGCs [9,12]. However, the integration of genome-based BGC prediction with experimental characterization of bioactive fractions from sandstorm-derived *Bacillus* strains remains limited. Therefore, the aim of this study was to integrate genome mining with phenotypic antimicrobial, siderophore, and biosurfactant assays, followed by bioactivity-guided fractionation and LC–MS/MS analysis of active fractions, to characterize the biosynthetic and antimicrobial potential of *Bacillus* strains (AD12–AD16) isolated from outdoor air during a sandstorm in Kuwait.

## 2. Results

### 2.1. Taxonomic Classification and Biosynthetic Gene Cluster Profiling of Bacillus Strains

Taxonomic classification assigned the environmental *Bacillus* strains in this study as *Bacillus stratosphericus* (AD12), *Bacillus safensis* (AD13), and *Bacillus subtilis* (AD14–16). Whole-genome mining using antiSMASH revealed that all five *Bacillus* strains (AD12–16) carried numerous BGCs that can code for the production of antibiotics, surfactants, and siderophores. One of the predicted BGCs in the WGS of AD12–13 was a bacillibactin BGC associated with siderophore production, and AD14–16 had a non-ribosomal peptide (NRP) BGC associated with surfactin and fengycin production (Table 1).

Moreover, several predicted BGCs showed no similarity to known reference BGCs in the antiSMASH database, identifying unassigned genomic regions with biosynthetic potential that warrant further characterization (Table 2).

### 2.2. Genome Assembly Quality

Whole-genome sequencing yielded high-quality draft genome assemblies for each of the five strains (Table 3). Genome size ranged from 3.64 Mb (AD12) to 4.26 Mb (AD15 and AD16), and GC content ranged from 41.31 to 43.68%. Sequencing depth ranged from 128× to 285×, which was sufficient for downstream genome mining analyses. The draft genome assemblies of the five *Bacillus* strains were deposited in the NCBI GenBank database under the accession numbers [JBWXEP000000000] (AD12), [JBSQAP000000000] (AD13), [JBSQAQ000000000] (AD14), [JBSQAR000000000] (AD15), and [JBSQAS000000000] (AD16).

RAST/RASTtk functional annotation revealed broadly similar functional profiles among the five *Bacillus* genomes. Approximately 1360–1585 genes were assigned to functional subsystems (Table S1). Functions associated with virulence, disease, and defense (58–75), resistance to antibiotics and toxic compounds (32–43), stress response (75–93), and iron acquisition and metabolism (35–44) were represented across the five genomes. Secondary metabolism-associated functions were also detected (26–39).

#### Antimicrobial Resistance Determinants and Mobile Genetic Elements

Analysis using CARD and ResFinder identified putative antimicrobial resistance determinants in all five strains. Two resistance-associated determinants were detected in AD12, four in AD13, three in AD14, four in AD15, and five in AD16. These included determinants associated with multidrug efflux and β-lactam resistance across the five strains, while putative bacitracin-associated resistance determinants were additionally identified in AD13, AD14, and AD15 (Table S2).

PlasmidFinder analysis identified putative plasmid replicons in three of the five *Bacillus* strains (Table S3). No known plasmid replicons were detected in AD12 or AD14, whereas one plasmid replicon was detected in each of AD13, AD15, and AD16. Mobile genetic element analysis identified insertion sequence- and transposon-associated elements in all five genomes, with five, seven, six, eight, and seven putative mobile genetic elements detected in AD12, AD13, AD14, AD15, and AD16, respectively.

### 2.3. Antimicrobial Activity of Bacillus Strains Against Indicator Bacteria

According to agar-well diffusion assays, the antimicrobial potential across all five *Bacillus* strains was statistically significant (*p* < 0.05). *B. stratosphericus* AD12 was able to inhibit *Escherichia coli* and *Staphylococcus aureus*. *B. safensis* AD13 displayed the broadest antimicrobial activity across all five *Bacillus* strains. It was able to inhibit all the indicator pathogens, including extensively drug-resistant (XDR) *Acinetobacter baumannii*, *Pseudomonas aeruginosa*, and *Klebsiella pneumoniae*, as shown by significantly larger zones of inhibition compared to AD12 (*p* < 0.05) (Table 4). The antimicrobial activity of *B. subtilis* (AD16) significantly exceeded the activity of *B. subtilis* AD14 and AD15 according to the zones of inhibition (*p* < 0.05).

The cell-free supernatants (CFS) were tested for their heat stability to provide more insight into the chemical nature of the secondary metabolites. After treatment with protease and heat, the CFS from AD12 and AD13 remained active, with no significant difference in antibacterial activity (*p* > 0.05). This confirms that the siderophores in the CFS are not proteinaceous and are heat-stable. In contrast, the CFSs of AD14–16 were stable after heat treatment, but significantly lost their antimicrobial activity after proteinase K treatment (*p* < 0.05). This finding suggests that protease-sensitive components might be involved in the antimicrobial activity of the crude supernatant.

### 2.4. Biosurfactant and Siderophore Production

**Figure 1:**
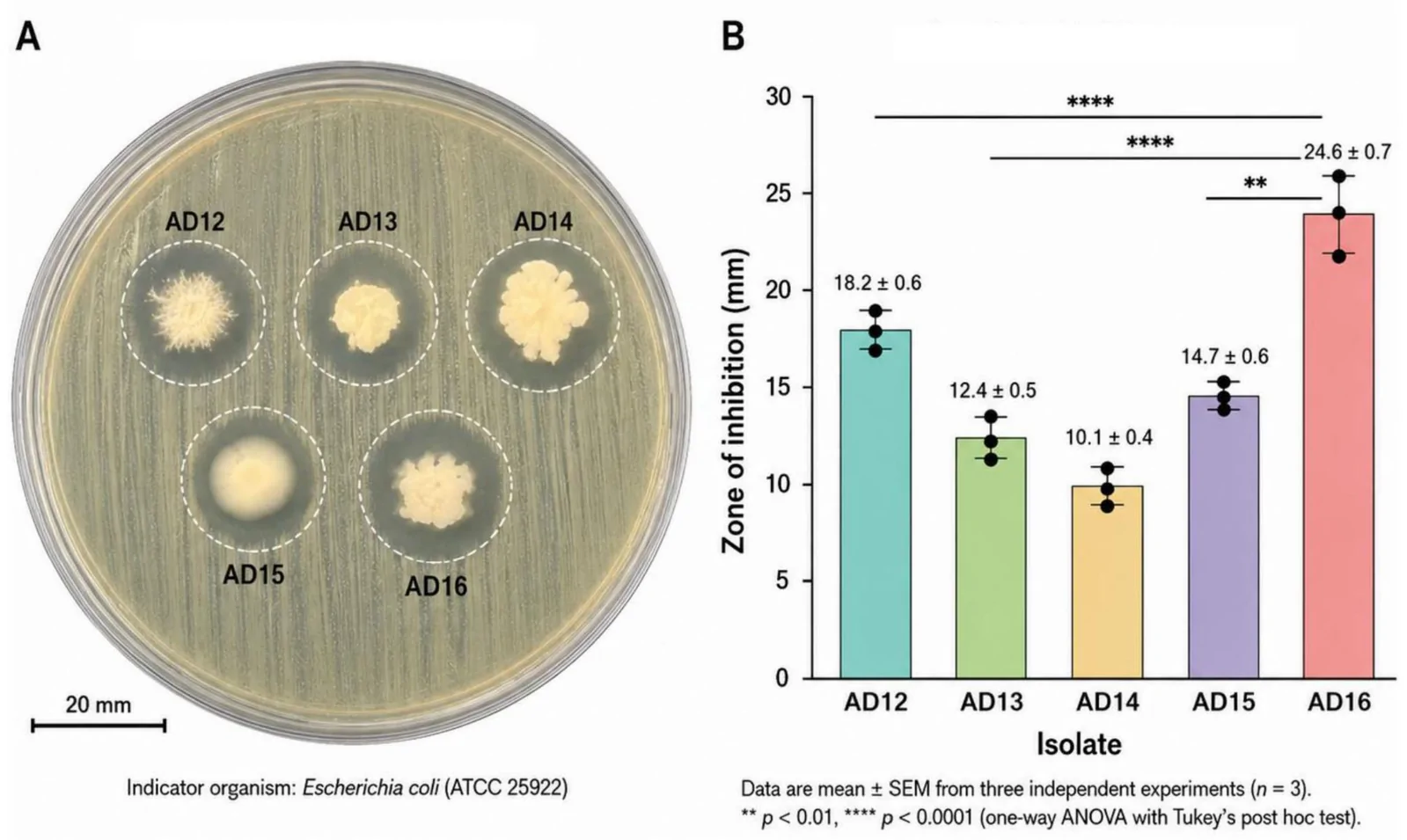
Antimicrobial activity of sandstorm-derived *Bacillus* strains against *E. coli.* (**A**) Representative spot-on-lawn assay demonstrating the antimicrobial activity of strains AD12–AD16 against *Escherichia coli* ATCC 25922. Clear zones surrounding the bacterial spots indicate inhibition of indicator organism growth. Scale bar = 20 mm. (**B**) Quantitative inhibition zone measurements obtained from the spot-on-lawn assay. Bars represent the mean zone of inhibition (mm) ± SEM from three independent experiments (*n* = 3). Statistical analysis was performed using one-way ANOVA followed by Tukey’s multiple comparisons test. AD16 exhibited the largest inhibition zone against *E. coli*, whereas AD14 showed the lowest antimicrobial activity.

All five *Bacillus* strains from the sandstorm showed antimicrobial activity against *E. coli* in the spot-on-lawn assay (Figure 1A). All strains showed clear inhibition zones, but the size of the activity varied significantly among the strains (*p* < 0.01). The highest antibacterial activity was found in *B. subtilis* AD16, with the largest inhibition zone (24.6 ± 0.7 mm), while AD14 showed the lowest activity (10.1 ± 0.4 mm). Intermediate inhibition was seen for AD12 (18.2 ± 0.6 mm), AD15 (14.7 ± 0.6 mm), and AD13 (12.4 ± 0.5 mm) (Figure 1B). AD13 exhibited the broadest antimicrobial spectrum, showing activity against all five indicator organisms, with mean inhibition zones ranging from 18.5 ± 0.3 mm against *A. baumannii* to 22.2 ± 0.8 mm against *P. aeruginosa*. Against *S. aureus*, AD16 produced an inhibition zone of 23.2 ± 0.2 mm compared with 21.2 ± 0.5 mm for AD15 and 16.4 ± 0.9 mm for AD14, corresponding to absolute differences of 2.0 and 6.8 mm, respectively. Among the *B. subtilis* isolates, AD16 showed the strongest activity against *E. coli*, with a mean inhibition zone of 24.1 ± 0.3 mm, compared with 20.0 ± 0.9 mm for AD15 and 17.0 ± 0.7 mm for AD14. This represented absolute differences of 4.1 and 7.1 mm relative to AD15 and AD14, respectively.

**Figure 2:**
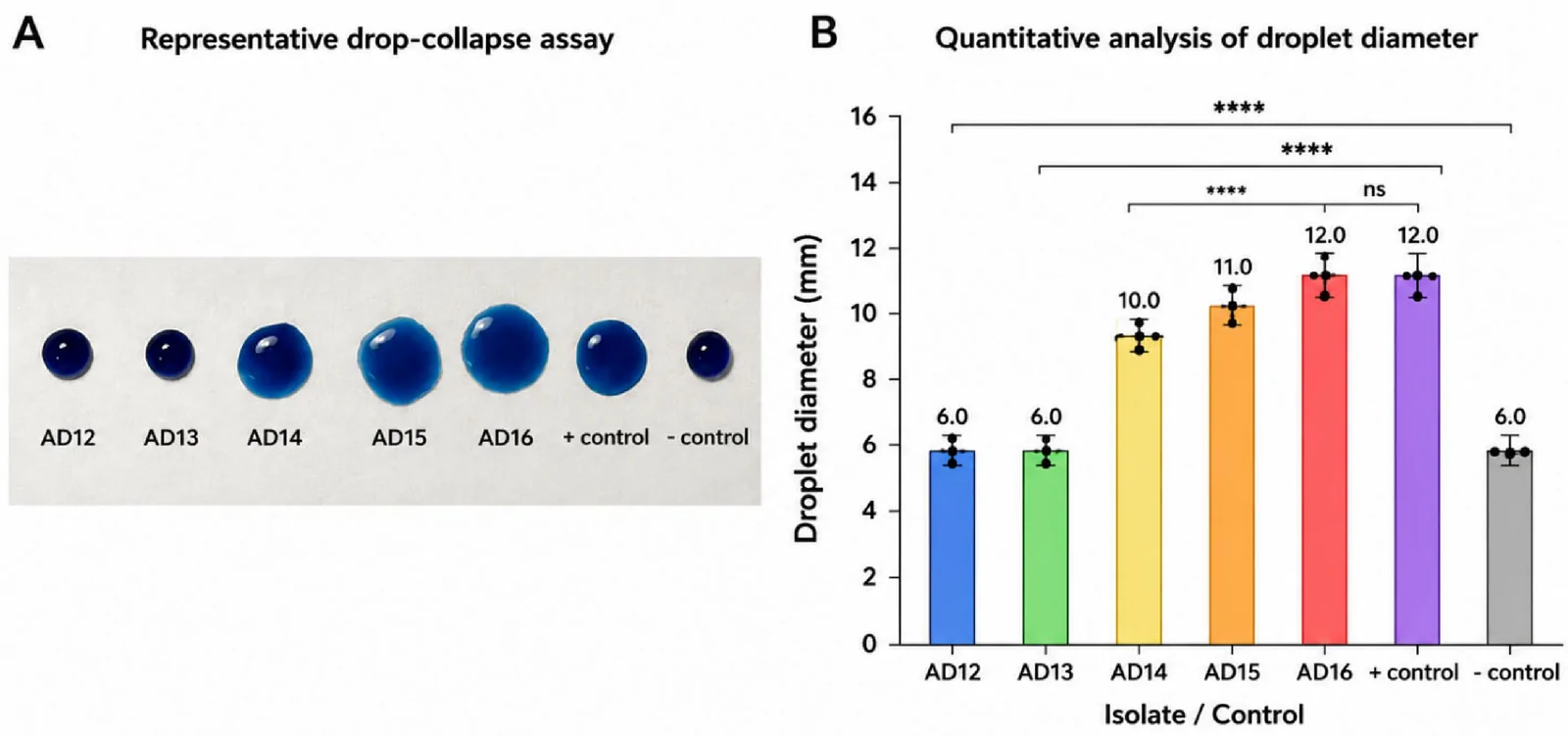
Biosurfactant activity of sandstorm-derived *Bacillus* strains determined using the drop-collapse assay. (**A**) Representative drop-collapse assay showing the spreading behavior of CFSs obtained from strains AD12–AD16. Increased droplet spreading is indicative of biosurfactant production. Positive and negative controls are included for comparison. (**B**) Quantitative analysis of droplet diameter. Bars represent the mean droplet diameter (mm) ± SEM from three independent experiments (*n* = 3). Droplet diameters were measured directly from the drop-collapse assay, with larger diameters indicating stronger biosurfactant activity. Statistical analysis was performed using one-way ANOVA followed by Tukey’s multiple comparisons test. Significant differences are indicated as follows: **** *p* < 0.0001; ns, not significant.

Among the sandstorm-derived *Bacillus* strains, biosurfactant production was significantly different (*p* < 0.0001) (Figure 2B). The *B. subtilis* strains AD14, AD15, and AD16 showed pronounced biosurfactant-associated activity, with median drop-collapse diameters of 10, 11, and 12 mm, respectively, compared with 6 mm for the negative control. These corresponded to absolute increases of 4, 5, and 6 mm, respectively. In contrast, AD12 and AD13 both showed a drop diameter of 6 mm, equivalent to the negative control.

**Figure 3:**
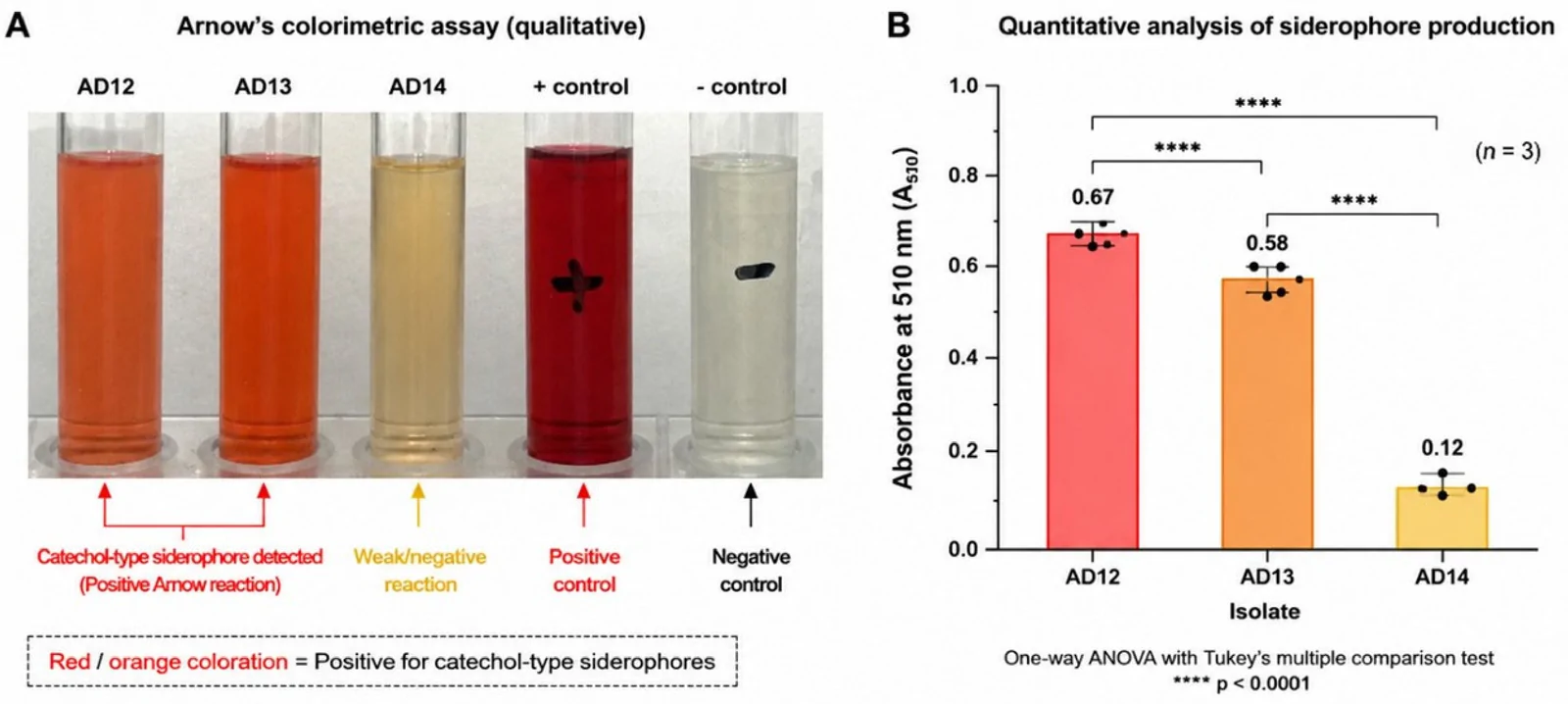
Detection and quantification of catechol-type siderophore production by sandstorm-derived *Bacillus* strains. (**A**) Representative Arnow’s assay showing catechol-type siderophore production. Red/orange coloration indicates a positive reaction. Positive and negative controls are shown for comparison. (**B**) Quantitative siderophore production measured as absorbance at 510 nm (A510). Bars represent mean ± SEM from three independent experiments (*n* = 3). Higher absorbance values indicate greater siderophore production. Statistical significance was determined using one-way ANOVA with Tukey’s multiple comparisons test.

Arnow’s assay confirmed catechol-type siderophore production in *B. stratosphericus* AD12 and *B. safensis* AD13, which produced significantly higher absorbance values at 510 nm (0.67 ± 0.02 and 0.58 ± 0.03, respectively) than *B. subtilis* AD14 (0.12 ± 0.01; *p* < 0.0001) (Figure 3B). The positive red–orange coloration observed for AD12 and AD13 further supported their siderophore-producing capability (Figure 3A).

### 2.5. Quantitative Antimicrobial Activity of Active Fractions

Active fractions obtained after extraction and fractionation of the CFSs were used for further evaluation of antimicrobial potency by minimum inhibitory concentration (MIC) and minimum bactericidal concentration (MBC) determination using representative multidrug-resistant pathogens. Fraction F1 inhibited *E. coli* and *K. pneumoniae* with MICs of 32 μg/mL and MBCs of 64 μg/mL (Table 5). Fraction F2 exhibited activity against *A. baumannii* and *P. aeruginosa* at 64 μg/mL (MIC) and 128 μg/mL (MBC), respectively. For all active fractions, MBC values were one dilution higher than the corresponding MIC values, resulting in MBC/MIC ratios of 2.

### 2.6. LC–MS/MS Profiling of Active Fractions

**Figure 4:**
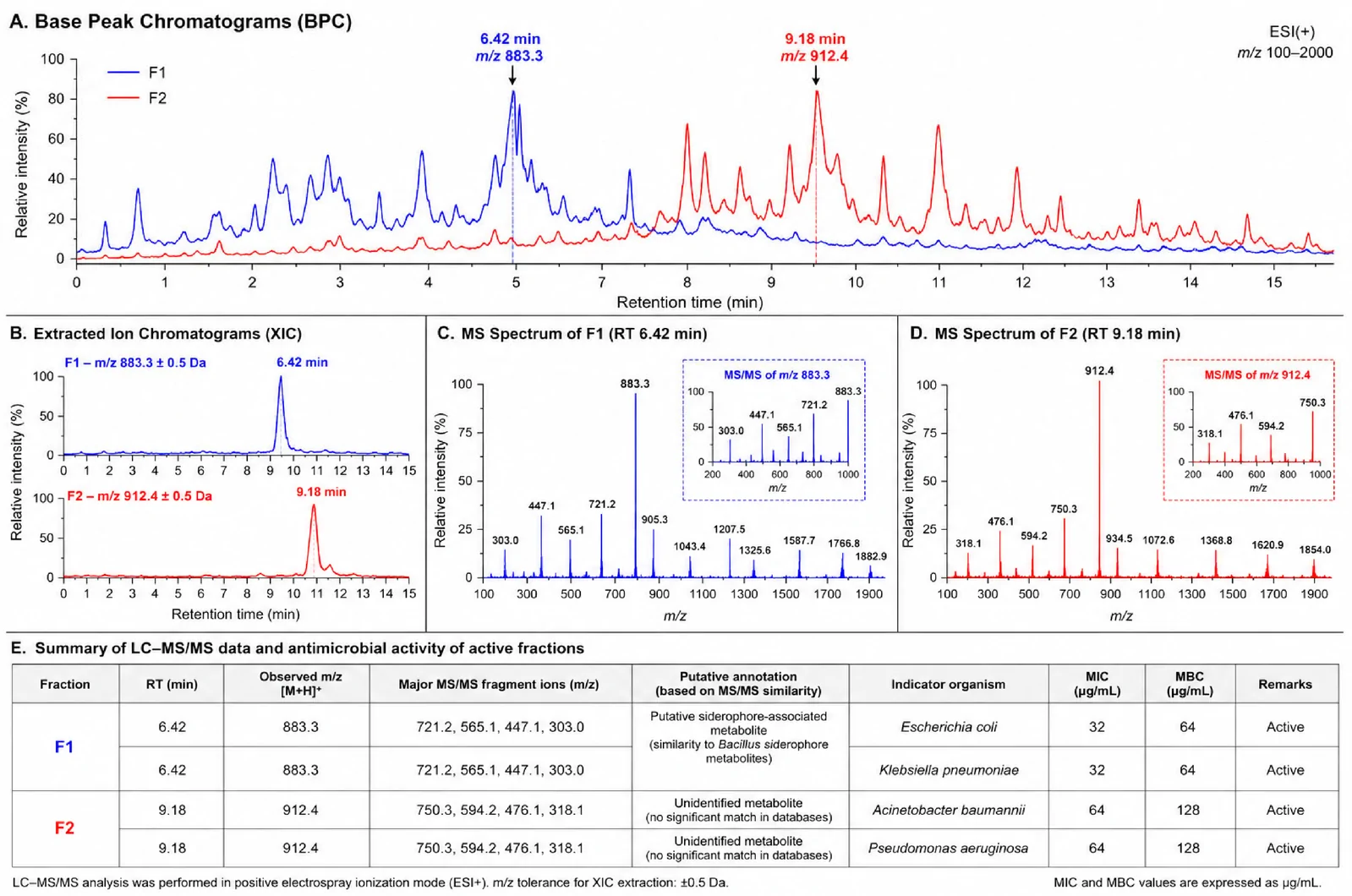
LC–MS/MS characterization of antimicrobial fractions F1 and F2 from *Bacillus safensis* AD13. Base peak chromatograms (**A**), extracted ion chromatograms (**B**), mass spectra, and MS/MS fragmentation patterns (**C**) and (**D**) of the active fractions are shown. The dominant ions detected were *m*/*z* 883.3 (RT 6.42 min) in F1 and *m*/*z* 912.4 (RT 9.18 min) in F2. F1 contained a molecular feature tentatively assigned to a siderophore-associated metabolite class and showed activity against *E. coli* and *K. pneumoniae*, whereas the dominant molecular feature in F2 remained unassigned, and the fraction showed activity against *A. baumannii* and *P. aeruginosa*. MIC and MBC values are summarized in panel (**E**). All metabolite annotations are tentative and based on LC–MS/MS data; definitive structural identification requires further purification and orthogonal characterization.

The active fractions were analyzed by liquid chromatography–tandem mass spectrometry (LC–MS/MS), revealing several molecular features that were tentatively assigned to known classes of *Bacillus* secondary metabolites based on accurate mass and MS/MS fragmentation patterns (Table 5). Fraction F1 exhibited inhibitory activity against *E. coli* and *K. pneumoniae* with MIC and MBC values of 32 μg/mL and 64 μg/mL, respectively. LC–MS/MS analysis of F1 showed a major ion at *m*/*z* 883.3, which was tentatively assigned to a siderophore-associated metabolite class based on comparison of its accurate mass and MS/MS fragmentation pattern with previously reported *Bacillus* metabolites. Antimicrobial activity of Fraction F2 was observed against *A. baumannii* and *P. aeruginosa* with MIC and MBC values of 64 μg/mL and 128 μg/mL, respectively. LC–MS/MS analysis revealed a dominant ion at *m*/*z* 912.4 (Figure 4). This molecular feature could not be confidently matched to a known metabolite in the databases examined and therefore remained unassigned.

## 3. Discussion

*Bacillus* species are well-known dominant members of dust-associated microbiota due to their spore-forming ability, resistance to UV radiation, and their ability to withstand extreme temperatures [3]. Unlike conventionally studied soil-derived *Bacillus* strains, airborne sandstorm-associated populations experience repeated cycles of atmospheric transport, desiccation, UV exposure, and nutrient limitation. These environmental pressures may influence microbial competitiveness, stress adaptation, and secondary metabolite biosynthesis [12]. The current study provides a genotypic and phenotypic glimpse of the antibacterial potential of five *Bacillus* strains isolated from airborne dust during a sandstorm in Kuwait. While several predicted BGCs were consistent with the observed antimicrobial, biosurfactant, and siderophore-associated phenotypes, the identification of multiple unassigned BGCs and bioactive fractions containing incompletely characterized metabolites represents an important finding warranting further investigation.

In addition to endospore formation, extracellular matrix production and multicellular organization may provide *Bacillus* populations with additional protection against environmental stresses. Biofilm-associated cells embedded within an extracellular matrix can exhibit increased tolerance to desiccation and other physicochemical stresses, while phenotypic heterogeneity within *Bacillus* communities allows different subpopulations to specialize in functions such as matrix production, sporulation, motility, and secondary metabolism [13]. Such division of labor could be advantageous during atmospheric transport, where dust-associated microorganisms are exposed to severe fluctuations in hydration, temperature, nutrient availability, and ultraviolet radiation [13]. In this context, the diverse secondary metabolite biosynthetic potential observed in the sandstorm-derived isolates may reflect broader ecological adaptations to environmental stress and microbial competition. However, biofilm formation, extracellular matrix production, and division of labor were not experimentally investigated in AD12–AD16; therefore, their contribution to survival during sandstorm transport remains to be determined.

Using antiSMASH, the WGS of all five *Bacillus* strains carried siderophore, NRPS, and PKS BGCs that showed high similarity and were well characterized. These known BGCs included those that code for the production of fengycin, bacilysin, bacillibacin, and pulcherriminic acid. Consistent with previous findings from genomic analyses, *Bacillus* spp. have been shown to harbor a diverse range of BGCs, most of which can code for the production of SMs with antimicrobial potential [12]. Interestingly, in our study, some predicted BGCs showed no similarity to known clusters in the antiSMASH/MiBiG database. The detection of these unassigned BGCs identifies genomic regions of particular interest for future characterization; however, their products and biological functions cannot be inferred from antiSMASH prediction alone. Under standard laboratory conditions, some BGCs remain cryptic or “silent”, but they can be activated through techniques including co-culture, ribosomal engineering, and environmental stresses [4]. Other studies on antibiotic-producing *Bacillus* strains have documented the activation of cryptic SM BGCs under stress conditions, leading to active antibiotic production [6]. Although antiSMASH is a powerful genome mining platform for predicting biosynthetic gene clusters, computational prediction alone cannot confirm metabolite production or structural novelty without experimental validation such as transcriptomics or LC–MS/MS analysis [5].

The present study identified *B. safensis* AD13 and *B. subtilis* AD16 as the most bioactive strains based on both diffusion-based screening assays and quantitative antimicrobial evaluation. While agar diffusion assays initially demonstrated broad inhibitory activity, subsequent MIC and MBC analyses provided a more rigorous assessment of antimicrobial potency. Active fractions obtained from AD13 exhibited activity against multiple multidrug-resistant Gram-negative pathogens, including *E. coli*, *K. pneumoniae*, *A.baumannii*, and *P. aeruginosa*.

The close agreement between MIC and MBC values observed for all active fractions (MBC/MIC ≤ 2) suggests that the bioactive metabolites exert bactericidal effects rather than merely inhibiting bacterial growth. These findings are consistent with previous reports demonstrating the production of potent antimicrobial metabolites by environmental *Bacillus* species, including lipopeptides, polyketides, and ribosomally synthesized antimicrobial compounds [12].

LC–MS/MS analysis of the active fractions revealed molecular features that were tentatively assigned to known *Bacillus* secondary metabolite classes based on accurate mass and MS/MS fragmentation patterns. Molecular features detected in fractions obtained from AD16 were tentatively assigned to surfactin- and fengycin-associated lipopeptide classes, whereas F1 from AD13 contained a molecular feature tentatively assigned to a siderophore-associated metabolite class. The dominant molecular feature detected in F2 from AD13 could not be confidently assigned and remained uncharacterized. Of particular interest, the dominant ion at *m*/*z* 912.4 in F2 could not be confidently assigned to a known metabolite using the databases examined. Together with the antibacterial activity of F2 against *A. baumannii* and *P. aeruginosa*, this unresolved molecular feature represents a priority for activity-guided purification and structural characterization. These observations were generally consistent with the biosynthetic potential predicted by antiSMASH; however, all LC–MS/MS-based annotations should be regarded as tentative and do not constitute definitive structural identification.

Importantly, although the antimicrobial activity and metabolomic profiles were broadly consistent with the predicted biosynthetic gene clusters, genome mining and LC–MS/MS analyses alone cannot establish a direct causal relationship between specific BGCs and the observed bioactivities. Definitive chemical identification will require activity-guided purification followed by structural characterization using approaches such as high-resolution MS/MS, comparison with authentic standards, and nuclear magnetic resonance (NMR) spectroscopy. Targeted gene-expression and mutagenesis studies would additionally be required to establish direct links between specific BGCs and the detected bioactive metabolites. The detection of several unidentified molecular features within AD13 further suggests the presence of unexplored biosynthetic potential.

After heat treatment and proteinase K treatment, antibacterial activity of AD12 and AD13 CFSs remained unchanged. This suggests that the antibacterial SMs in the CFS are non-proteinaceous, which can include polyketides and siderophores. This supports the antiSMASH findings, which predicted the presence of bacillibactin and schizokinen-like siderophore BGCs, both of which are heat-stable and resist proteolytic digestion [14]. In contrast, the CFSs of *B. subtilis* AD14, AD15, and AD16 remained stable after heat treatment but lost their antibacterial activity following treatment with proteinase K.

Although antiSMASH predicted BGCs with similarity to surfactin- and fengycin-associated pathways, surfactin and fengycin are cyclic lipopeptides and are generally resistant to many proteases. Therefore, loss of activity after proteinase K treatment does not directly confirm degradation of surfactin or fengycin. The reduction in activity may indicate the presence of protease-sensitive peptide components, peptide-associated bioactive metabolites, or complex interactions within the crude supernatant [12]. Further purification, LC–MS/MS profiling, and activity-guided fractionation are required to determine the exact compounds responsible for the antimicrobial activity.

Biosurfactant activity was strongly detected in all *B. subtilis* strains (AD14–16), which aligns with antiSMASH results that predicted surfactin and fengycin BGCs. Surfactin and fengycin are two potent lipopeptide biosurfactants produced by *Bacillus* spp. [15]. As demonstrated by several studies, the presence of surfactants has been correlated with large collapse diameters in drop-collapse assays, which makes this assay a reliable indicator of biosurfactant production [16]. The strong biosurfactant-associated activity observed in AD14–AD16 is consistent with the known capacity of *B. subtilis* to produce surface-active metabolites. However, the present study did not assess whether production of these metabolites contributes specifically to survival during atmospheric or sandstorm transport [15]. In sandstorm environments, which are harsh and particulate-rich, secretion of biosurfactants by *B. subtilis* can provide a survival advantage.

Siderophores function as iron chelators and play a role in competitive interactions, depriving other bacteria of iron and ultimately inhibiting their growth [16]. In this study, siderophore production was only detected in *B. stratosphericus* and AD12 and *B. safensis* AD13, which correlates with antiSMASH findings that predicted multiple siderophore BGCs in the WGS of both strains. *B. stratosphericus* was previously isolated from high-altitude environments where bioavailable iron levels are low, creating strong selective pressure for bacteria capable of synthesizing siderophores [17]. The strong siderophore activity displayed by AD12 and AD13 demonstrates iron-chelating capability under the conditions tested; however, its specific contribution to survival during atmospheric transport was not examined in the present study. In sandstorm environments in particular, microorganisms can experience harsh conditions including UV exposure, nutrient scarcity, and desiccation, triggering the activation of iron-chelating systems. A recent study reported that dust-associated *Bacillus* populations have multiple siderophore biosynthetic pathways, reflecting their adaptation to nutrient-limited and transient niches [18]. Thus, AD12 and AD13 represent bioactive airborne *Bacillus* strains with valuable SM production capability. Importantly, the observed phenotypic differences were also of appreciable biological magnitude. AD13 exhibited the broadest antimicrobial spectrum, with activity against all five indicator organisms and inhibition zones ranging from 18.5 ± 0.3 to 22.2 ± 0.8 mm. Among the *B. subtilis* isolates, AD16 showed particularly strong antimicrobial activity, including an inhibition zone of 24.1 ± 0.3 mm against *E. coli*, representing a 7.1 mm greater mean inhibition zone than AD14. Biosurfactant-associated responses were also pronounced in AD14–AD16, with median drop-collapse diameters of 10–12 mm compared with 6 mm for the negative control. In contrast, AD12 and AD13 showed strong catecholate-associated responses, with A510 values of 0.67 ± 0.02 and 0.58 ± 0.03, respectively. Collectively, the magnitude and consistency of these phenotypic responses provide biological context for the statistical comparisons and support the biological relevance of the observed differences.

Despite the promising antimicrobial activity observed in this study, the active fractions remain chemically complex mixtures, and the specific compounds responsible for antibacterial activity have not yet been purified. Furthermore, metabolite assignments were based primarily on accurate mass measurements and comparison with known metabolite databases. Consequently, the observed associations between predicted biosynthetic gene clusters, LC–MS/MS features, and antimicrobial phenotypes should be interpreted as supportive rather than confirmatory evidence [5,11]. Future studies involving activity-guided purification, transcriptomics, targeted gene knockout experiments, and structural characterization will be necessary to fully elucidate the molecular basis of the observed activities [6]. One limitation of the present study is the lack of detailed environmental metadata associated with the original sandstorm sampling event, including particulate matter concentrations, wind velocity, humidity, and aerosol physicochemical composition. Since the strains analyzed in this study were retrospectively selected from a previously archived airborne microbiology investigation, such environmental measurements were not available for downstream ecological correlation [8]. The biosynthetic capabilities of *Bacillus* species isolated from sandstorm-associated airborne dust are diverse and represent a promising source of bioactive secondary metabolites. However, the absence of detailed atmospheric metadata for the original sampling event limits interpretation of associations between specific environmental conditions and the observed microbial phenotypes. Future studies integrating atmospheric measurements with genomic and metabolomic analyses would provide deeper insight into the ecological adaptation and biosynthetic responses of airborne sandstorm-associated microorganisms [11]. Overall, the genotypic and phenotypic features of sandstorm-derived *Bacillus* strains in this study demonstrate their biosynthetic versatility. Sandstorm-derived *Bacillus* strains represent an underexplored reservoir of biosynthetic diversity that warrants further metabolomic and functional investigation.

## 4. Materials and Methods

### 4.1. Origin of Bacillus Strains

*Bacillus* isolates used in this study were previously recovered from airborne dust samples collected from an open-air sampling site at Mubarak Al-Kabeer Hospital, Kuwait (29°19′34″ N, 48°02′06″ E), during an active sandstorm in May 2021, as part of a previous environmental airborne microbiology monitoring study [8]. Air sampling was done triplicate outdoors using an SKC Biostage™ impactor air sampler (SKC Inc., Eighty Four, PA, USA). Air sampling was performed in triplicate outdoors using an SKC BioStage™ single-stage viable cascade impactor (SKC Inc., Eighty Four, PA, USA) on nutrient agar and R2A agar (Sigma–Aldrich, St. Louis, MO, USA) supplemented with vancomycin (10 µg/mL), ciprofloxacin (10 µg/mL), cycloheximide (50 µg/L) and nystatin (10 µg/L) to suppress fungal growth and enrich for environmentally resistant bacterial populations. Plates were incubated at 22 °C for 3–4 days, and visible colonies were selected and stored at −70 °C.

The present study focused on downstream phenotypic and genomic characterization of previously archived *Bacillus* strains recovered from a sandstorm-associated sampling event. For the original sampling survey, detailed atmospheric metadata such as concentrations of particulate matter (PM10/PM2.5), wind velocity, humidity, temperature fluctuations, and aerosol physicochemical composition were not available and therefore could not be analyzed retrospectively. The isolates, however, are representative of the airborne *Bacillus* populations recovered during documented sandstorm conditions in Kuwait, an environment of frequent dust events, high particulate loads, desiccation stress, and high ultraviolet exposure.

We analyzed 16S rRNA from single isolated colonies from each antibiotic plate. Amplifications were performed in a 50 µL reaction volume containing 1–2 µL of lysed cell sample, 5X FIREPol Master Mix, and 10 µM of each primer, 27F (AGAGTTTGATCCTGGCTCAG) and 1492R (GGTTACCTTGTTACGACTT). The PCR program consisted of 5 min at 95 °C, 35 cycles of 30 s at 94 °C, 30 s at 54 °C, and 2 min at 72 °C, followed by 1 cycle of 10 min at 72 °C. The PCR products were separated on a 1% agarose gel and purified using the QIAQuick PCR Purification kit. The PCR amplicons were then sequenced. Identification at the species level was performed by comparison with the Ribosomal Database Project database (http://rdp.cme.msu.edu/) (accessed on 1 March 2026) and by using BLAST (http://blast.ncbi.nlm.nih.gov/Blast.cgi, accessed on 1 March 2026).

From the archived collection, five isolates (AD12–AD16) were selected for the present study based on their preliminary identification as members of the genus *Bacillus*. The selection was not based on antimicrobial activity, as antimicrobial screening was performed subsequently as part of the present study. These isolates were then subjected to phenotypic and whole-genome analyses to characterize their antimicrobial and biosynthetic potential. Details of whole-genome sequencing and bioinformatic analyses are provided in Section 4.13.

From the pool of identified strains, five *Bacillus* strains were selected and subjected to subsequent phenotypic and genomic analyses in this study. Five *Bacillus* strains (AD12, 13, 14, 15 and 16) were subcultured onto nutrient agar and were preliminarily identified using Gram staining and biochemical tests. Identification at the species level was verified by whole-genome sequencing and average nucleotide identity (ANI) analysis.

### 4.2. Test Microorganisms

In the present study, the antimicrobial spectrum of the five *Bacillus* strains was assayed using pathogenic bacteria as indicator strains. The indicator bacteria were *S. aureus*, *P. aeruginosa*, *E. coli*, *K. pneumoniae,* and *A. baumannii* (Table 6). Briefly, these indicator bacteria were cultured on nutrient agar at 37 °C for 24 h to obtain single colonies. Then, each indicator was cultured in a suspension by adding one colony to 10 mL of nutrient broth for 18 h while shaking at 150 rotations per minute (rpm).

### 4.3. Spot-on-Lawn Assay

The spot-on-lawn assay was performed as previously described [19]. All indicator bacteria were grown overnight in nutrient broth, while the 5 *Bacillus* strains were grown overnight in Tryptone Soy broth (TSB) (Oxford, UK). The indicator bacterial cultures were adjusted to a 0.5 McFarland standard and were spread onto Mueller–Hinton agar plates (Sigma–Aldrich, St. Louis, MO, USA) to create a uniform lawn of bacteria. Onto this lawn, 5 µL of *Bacillus* culture was gently spotted and allowed to air-dry before incubation at 37 °C for 24 h. The presence of a clear halo around each *Bacillus* spot was recorded as positive antibacterial activity.

### 4.4. Preparation of Cell-Free Supernatants

One colony of each *Bacillus* strain (AD12–16) was grown in 20 mL TSB at 35 °C for 24 h while shaking at 150 rpm. At each time point, the cultures were centrifuged for 30 min at 15,000 rpm to separate the supernatant from the bacterial pellet. CFSs were prepared by collecting supernatants and passing them through 0.22 µM membrane filters [20].

### 4.5. Detection of Antibacterial Activity Using Agar-Well Diffusion Assays

The CFSs were evaluated for antibacterial activity against the indicator bacteria using agar-well diffusion assays [21]. The indicator bacteria were prepared and adjusted to a 0.5 McFarland standard and spread as a lawn onto Mueller–Hinton agar plates. Wells of 8 mm diameter were prepared by punching wells using 1 mL sterile disposable pipette tips. These wells were filled with CFS (100 µL) and were allowed to rest at room temperature to allow full absorption into the agar wells. These plates were incubated overnight at 37 °C, and subsequently, the diameters of zones of inhibition were measured (in mm). No inhibitory activity was reported when the inhibition zone measured 8 mm. This experiment was conducted in triplicate.

### 4.6. Detection of Biosurfactants Using Drop-Collapse Assays

To test the presence of biosurfactants produced by all five *Bacillus* strains, a drop-collapse assay was performed using the protocol described previously with minor alterations [22]. Briefly, one drop of distilled water (25 μL) was pipetted onto a hydrophobic surface, followed by a drop of methylene blue (10 μL). The CFS (25 μL) was pipetted carefully onto this droplet, and the drop diameter was measured after collapse (in mm). An increase in drop size compared to the negative control (sterile nutrient broth instead of CFS) was due to the presence of surfactants. A positive control (0.1% Triton X-100) was used in this assay. A collapsed droplet was reported when the diameter was at least 1 mm larger than that of the negative control [22]. This experiment was conducted in triplicate.

### 4.7. Detection of Siderophore Production Using Arnow’s Test

Arnow’s test was used to detect the presence of catechol groups in siderophores in the CFS [23]. The CFS (1 mL) was mixed with 0.5 mL of HCL (5 M), 0.1 mL of NaOH (10 N), and 1 mL of nitrite–molybdate reagent, followed by observation of color change. Catecholate siderophore production was confirmed if an orange–red color was observed. Using a FLUOstar Omega™ spectrophotometer (BMG LABTECH GmbH, Ortenberg, Germany), the absorbance of each reaction was measured at 510 nm (A510). This experiment was conducted in triplicate.

### 4.8. CFS Treatment with Proteinase K

To determine whether antimicrobial activity was due to proteins or peptides, the CFS was treated with proteinase K (Sigma–Aldrich, St. Louis, MO, USA) at a concentration of 10 mg/mL [24]. The CFS was incubated for 1 h at 37 °C with the enzyme, followed by inactivation by the addition of phenylmethylsulfonyl fluoride. The proteinase K-treated CFS was then assessed for antibacterial activity using agar-well diffusion assays. If antimicrobial activity was reduced after treatment, this suggested that the active compound/s in the CFS were proteinaceous in nature.

### 4.9. Thermal Stability of CFS

To test if the antimicrobial activity was heat-stable, the CFS was exposed to 70 °C for 30 min using a water bath (Thermo Fisher Scientific, Waltham, MA, USA). The heat-treated CFS was then assessed for antibacterial activity using agar-well diffusion assays [25]. If antimicrobial activity was retained after heat treatment, this suggested that the active compound/s in the CFS were heat-stable.

### 4.10. Extraction and Fractionation of Metabolites from Bacillus safensis AD13

Based on the antimicrobial screening results, *B. safensis* AD13 was selected for further investigation since it exhibited the broadest spectrum of antimicrobial activity against multidrug-resistant indicator organisms. In addition, AD13 displayed positive siderophore activity and had several biosynthetic gene clusters predicted by antiSMASH, including siderophore-related and unassigned clusters, indicating biosynthetic potential.

For metabolite extraction, AD13 was cultured in 100 mL TSB at 35 °C for 24 h with shaking at 150 rpm. Cultures were centrifuged at 10,000× *g* for 20 min, and the resulting CFSs were passed through sterile 0.22 μm membrane filters. The filtered CFS was acidified to pH 2.0 using 6 M hydrochloric acid and incubated overnight at 4 °C to precipitate extracellular metabolites [26]. The precipitates were collected by centrifugation at 12,000× *g* for 30 min, washed with acidified distilled water (pH 2.0), and resuspended in methanol. Methanolic extracts were evaporated under reduced pressure and reconstituted in 1 mL methanol.

Crude extracts were fractionated using a reversed-phase C18 solid-phase extraction (SPE) column (Sigma–Aldrich, St. Louis, MO, USA) [27]. Elution was performed sequentially using increasing concentrations of methanol in water (20%, 40%, 60%, and 100%). Fractions displaying antimicrobial activity were designated as active fractions and selected for subsequent antimicrobial and metabolomic analyses. Positive control antibiotics were selected according to the known susceptibility profile of each indicator organism to ensure measurable inhibitory activity. As a negative extraction control, uninoculated TSB was incubated and processed in parallel under the same conditions as the AD13 culture. The corresponding control fractions were tested using the same antimicrobial assay and showed no detectable inhibitory activity against the indicator organisms. A solvent-only control containing the corresponding volume of methanol was also tested and showed no inhibitory activity. These controls were included to exclude antimicrobial effects arising from medium-derived components or the extraction solvent.

### 4.11. Determination of MIC and MBC

The minimum inhibitory concentration (MIC) and minimum bactericidal concentration (MBC) of the purified fractions were determined using the broth microdilution method according to CLSI guidelines [28]. Briefly, two-fold serial dilutions ranging from 1 to 256 μg/mL were prepared and inoculated with approximately 5 × 10^5^ CFU/mL of each indicator organism. Plates were incubated at 37 °C for 24 h. The MIC was defined as the lowest concentration showing no visible growth. To determine MBC values, aliquots from wells showing no visible growth were subcultured onto Mueller–Hinton agar and incubated for 24 h. The MBC was defined the lowest concentration resulting in no bacterial recovery.

### 4.12. LC–MS/MS Analysis of Active Fractions

Active fractions obtained from AD13 were analyzed using an Agilent 1290 Infinity II UHPLC system coupled to an Agilent 6545 Q-TOF mass spectrometer (Agilent Technologies, Santa Clara, CA, USA) equipped with an electrospray ionization source operating in positive ion mode [12]. Separations were carried out on a C18 reversed-phase column (2.1 × 100 mm, 1.8 μm particle size; Agilent Technologies, Santa Clara, CA, USA). Mobile phase A consisted of water containing 0.1% formic acid, and mobile phase B was acetonitrile containing 0.1% formic acid. Metabolites were separated using a linear gradient from 5% to 95% mobile phase B over 15 min at a flow rate of 0.3 mL/min. Mass spectra were recorded in the *m*/*z* range of 100–2000. Tentative metabolite annotation was performed by comparing accurate masses and MS/MS fragmentation patterns with previously reported *Bacillus* metabolites and publicly available metabolite databases [12]. All assignments were considered tentative, and definitive structural identification would require purification and orthogonal characterization, such as comparison with authentic standards and/or NMR spectroscopy.

### 4.13. Whole-Genome Sequencing and Assembly

Genomic deoxyribonucleic acid (DNA) was extracted using the QIAamp^®^ DNA Mini Kit (Qiagen, Hilden, Germany) and quantified using a NanoDrop-800 spectrophotometer (Thermo Fisher Scientific, Wilmington, NC, USA) according to the manufacturer’s instructions [29]. Whole-genome sequencing (Illumina MiSeq^®^) was carried out (Microbes NG, Birmingham, UK, https://microbesng.uk) (accessed on 1 September 2025). Raw sequencing reads were subjected to quality assessment using FastQC v0.11.9. Sequence reads were quality-trimmed using Trimmomatic [30]. Quality-filtered reads were assembled de novo using SPAdes v3.15.5. No additional standalone scaffolding step was performed following de novo genome assembly. Sequence processing and quality assessment were performed using the MicrobesNG bioinformatics pipeline incorporating SAMtools v1.9, BEDTools v2.29.2, and BWA-MEM v0.7.17 [31].

Genome assembly quality was evaluated using QUAST v5.0.2 [32] with assembly statistics calculated for contigs ≥500 bp. Genome completeness and contamination were additionally estimated using CheckM2 v1.0.2 [33,34]. Genome assemblies and annotations were examined using Artemis v18.2.0 [33]. Genome visualization was performed using the Proksee server (accessed on 1 September 2026), with genome annotation performed using Prokka v1.14.6 [35]. Species-level identification was confirmed using whole-genome sequencing and average nucleotide identity (ANI) analysis. Biosynthetic gene clusters were subsequently identified using antiSMASH as described in Section 4.15.

16S rRNA was identified using the SILVA 16S and Kraken databases revealed that strain AD12 was *Bacillus stratosphericus* (accession JBWXEP000000000), AD13 was *Bacillus safensis* (accession JBSQAP000000000), and strains AD14, AD15, and AD16 were identified as *Bacillus subtilis* with accession numbers JBSQAQ000000000, JBSQAR000000000, and JBSQAS000000000, respectively. The corresponding BioSample, Sequence Read Archive (SRA), GenBank WGS, and genome assembly accession numbers are provided in Table 3. Genome annotation statistics, including total genes, coding sequences (CDSs), protein-coding genes, rRNA genes, and pseudogenes, are also provided in Table 3.

#### 4.13.1. RAST Functional Annotation

Functional annotation of the five assembled genomes was additionally performed using the Rapid Annotation using Subsystem Technology (RAST) server with the RASTtk annotation scheme [36]. Functional subsystem distributions were examined with particular emphasis on genes associated with virulence and defense, resistance to antibiotics and toxic compounds, stress responses, iron acquisition and metabolism, and secondary metabolism. The functional profiles of the five strains were subsequently compared.

#### 4.13.2. Antimicrobial Resistance Genes and Mobile Genetic Elements Analysis

Antimicrobial resistance determinants were investigated using the Comprehensive Antibiotic Resistance Database (CARD v3.2.9) through the Resistance Gene Identifier (RGI v6.0.3) [37]. CARD results were interpreted according to the RGI Perfect and Strict detection categories, while Loose hits were excluded from the principal resistance gene analysis.

Acquired antimicrobial resistance genes were additionally investigated using ResFinder v4.3.3 [38]. ResFinder hits were considered positive when they met minimum thresholds of 90% sequence identity and 60% sequence coverage relative to the corresponding reference sequences. ResFinderFG v2.0 and KmerResistance v2.2 were additionally used as complementary approaches for resistance gene prediction.

Plasmid replicons were investigated using PlasmidFinder v2.1 [39]. A minimum sequence identity threshold of 95% and a minimum sequence coverage of 60% were used for plasmid replicon detection. Mobile genetic elements were investigated using MobileElementFinder v1.0.3 [40] to identify insertion sequences, transposons, integrative elements, and other mobile genetic elements and to examine their potential association with antimicrobial resistance determinants.

The pathogenic potential of the five isolates was assessed using PathogenFinder v1.1 [41]. PathogenFinder predictions were based on pathogenicity-associated protein families identified from the whole-genome sequences.

### 4.14. Genome Assembly Quality Assessment

Genome assemblies were assessed using QUAST for assembly statistics such as genome size, number of contigs, GC content, largest contig size, and N50 values. The trimmed reads were mapped to the assembled genomes using bwa-mem, SAMtools, and BEDTools to estimate the sequencing depth. Genome completeness and contamination were estimated using CheckM2 v1.0.2 [34]. The completeness and contamination values obtained for each of the five genome assemblies are presented in Table 3.

### 4.15. Biosynthetic Gene Cluster (BGC) Prediction

Biosynthetic genes that have the potential to code for the production of proteins involved in antimicrobial production were predicted by submitting the WGS of all 5 *Bacillus* strains (AD12–16) to antiSMASH (v7) [5]. antiSMASH was accessed through the website: https://antismash.secondarymetabolites.org/ (accessed on 4 April 2026). The sequence file in FASTA format was uploaded, with selection of well-defined clusters. The detection strictness was set to “relaxed” to permit more inclusive detection of putative BGC regions. This setting represents an algorithmic detection mode within antiSMASH and does not correspond to a fixed percentage identity or sequence coverage threshold. Predicted BGCs were compared with characterized reference BGCs in the MIBiG database, and the percentage similarity to known BGCs was recorded. The extra features, including KnownClusterBlast, MIBiG cluster comparison, Cluster Pfam analysis, TFBS analysis, ClusterBlast, ActiveSiteFinder, Pfam-based GO term annotation, NCBI genomic context links, SubClusterBlast, RREFinder, and TIGRFam analysis, were all enabled.

### 4.16. Statistical Analysis

Data are presented as mean ± standard deviation (SD) from three independent experiments. Statistical significance was defined as *p* < 0.05. In addition to statistical significance, absolute differences between group means and the consistency of responses across replicate experiments were considered when interpreting the magnitude and biological relevance of the observed phenotypic differences. The differences in antimicrobial activity, siderophore and surfactant production among the five *Bacillus* strains were analyzed using one-way ANOVA followed by Tukey’s post hoc test for multiple comparisons. Data were analyzed using Statistical Package for the Social Sciences (SPSS) Software version 24. Statistical significance was set at *p* < 0.05.

## 5. Conclusions

Sandstorm-derived *B. stratosphericus*, *B. safensis*, and *B. subtilis* strains showed diverse biosynthetic capability as demonstrated by the integration of genome mining and phenotypic assays in this study. The identification of multiple unassigned BGCs, together with the antimicrobial activity and LC–MS/MS profiles of active fractions from *B. safensis* AD13, highlights biosynthetic features that warrant further chemical and functional characterization. These strains demonstrated biosynthetic capability, including antimicrobial, siderophore, and biosurfactant production, which was complemented by the BGCs predicted by antiSMASH. Multiple unassigned BGCs were predicted by antiSMASH, identifying genomic regions with unexplored biosynthetic potential that require experimental characterization. The results show the biosynthetic potential of the strains and warrant further investigation. This study provides an integrated genomic and phenotypic characterization of airborne sandstorm-derived *Bacillus* strains recovered in Kuwait. Further metabolomic analyses and comparative genomic studies with publicly available genomes of closely related *Bacillus* species from diverse environmental sources will be needed to determine whether the unassigned BGCs identified in the present isolates are unique to or enriched in sandstorm-associated *Bacillus* populations and to investigate their potential functional relevance.

## Figures and Tables

**Table 1:** Overview of known BGCs detected in *Bacillus* strains AD12–16.

*Bacillus* Strain	Identified Known Clusters		
	Known BGCs Predicted by AntiSMASH	Major BGC Classes	Most Similar Known Clusters (Similarity)
*Bacillus stratosphericus* AD12	4	NRPS, RiPP-like, betalactone, NI-siderophore, terpene	Lichenysin (High), Bacilysin (High), Fengycin (Medium), Schizokinen-like siderophore (Medium)
*Bacillus safensis* AD13	7	NRPS, PKS/PKS-like, RiPPs (sacti/ranthi), betalactone, NI-siderophore	Bacilysin (High), Bacillibacin/Bacillibactin (High), Lichenysin (High), Sporulation killing factor (High), Fengycin (Medium), Schizokinen-like siderophore (Medium), Zwittermicin A (Low)
*Bacillus subtilis* AD14	8	NRPS, PKS (T3PKS, transAT-PKS), RiPPs, CDPS, terpenes	Fengycin (High), Surfactin (High), Bacillaene (High), Pulcherriminic acid (High), Subtilosin A (High), Bacillibactin (High), Tridecaptin (Low)
*Bacillus subtilis* AD15	6	NRPS, PKS, RiPPs, CDPS, betalactone, terpenes	Fengycin (High), Surfactin (High), Subtilosin A (High), Subtilin (High), Bacillibactin (High), Pulcherriminic acid (High), Bacilysin (High)
*Bacillus subtilis* AD16	6	NRPS, PKS, RiPPs, CDPS, betalactone, terpenes	Fengycin (High), Surfactin (High), Subtilosin A (High), Subtilin (High), Bacillibactin (High), Pulcherriminic acid (High), Bacilysin (High)

Similarity confidence according to known reference BGCs in the antiSMASH database (High = strong homology to a known cluster), (Medium = moderate similarity to known clusters), (Low = weak similarity to known cluster), BGC; biosynthetic gene cluster, NRPS; non-ribosomal peptide synthetase, T3PKS; Type 3 polyketide synthase, RiPP; Other unspecified ribosomally synthesized and post-translationally modified peptide product, RiPP-like; Other unspecified ribosomally synthesized and post-translationally modified peptide-like product, betalactone; Beta-lactone containing protease inhibitor, NI-siderophore; NRPS-independent, IucA/IucC-like siderophores.

**Table 2:** Overview of unassigned BGCs detected in *Bacillus* strains AD12–16.

*Bacillus* Strain	Unassigned BGCs	
	Unassigned BGCs Predicted by AntiSMASH	Major BGC Classes
*Bacillus stratosphericus* AD12	7	RiPP-like, T1PKS, Terpene, Terpene precursor, RRE-containing, Betalactone, RiPP-like
*Bacillus safensis* AD13	6	Betalactone, RiPP-like, Terpene-precursor, T3PKS, RRE-containing, terpene
*Bacillus subtilis* AD14	6	Terpene, Terpene-precursor, epipeptide, terpene, NRPS, NRPS-like
*Bacillus subtilis* AD15	5	terpene-precursor, terpene, terpene, epipeptide, RiPP-like
*Bacillus subtilis* AD16	5	terpene-precursor, terpene, terpene, epipeptide, RiPP-like

Unassigned BGCs are clusters showing no similarity to known reference BGCs in the antiSMASH database, indicating potential for novel secondary metabolite production. RiPP-like; Other unspecified ribosomally synthesized and post-translationally modified peptide product (RiPP), RRE-containing; RRE-element containing cluster, NRPS; Non-ribosomal peptide synthetase, Terpene-precursor; Compound likely used as a terpene precursor, epipeptide; D-amino-acid containing RiPPs.

**Table 3:** Genome assembly statistics of sandstorm-derived *Bacillus* strains.

Feature	AD12	AD13	AD14	AD15	AD16
Species	*B. stratosphericus*	*B. safensis*	*B. subtilis*	*B. subtilis*	*B. subtilis*
Genome size (Mb)	3.64	3.70	4.09	4.26	4.26
GC (%)	41.31	41.66	43.68	43.38	43.38
No. contigs	27	57	25	17	38
Genome completeness (%)	99.74	99.12	99.78	98.83	98.88
Contamination (%)	0.35	0.41	0.41	0.18	0.25
Total genes	3819	3819	4214	4392	4387
Total CDSs	3716	3716	4132	4309	4304
Protein-coding genes	3689	3689	4049	4224	4219
rRNA genes	6	10	12	12	11
Pseudogenes	27	27	71	73	74
BioProject accession	PRJNA1370681	PRJNA1371112	PRJNA1371115	PRJNA1371116	PRJNA1371118
BioSample accession	SAMN53475619	SAMN53489894	SAMN53489940	SAMN53489941	SAMN53489986
GenBank WGS accession	JBWXEP000000000	JBSQAP000000000	JBSQAQ000000000	JBSQAR000000000	JBSQAS000000000
Genome assembly accession	GCA_056679755.1	GCA_056679755.1	GCA_060334745.1	GCA_060334765.1	GCA_060334785.1

Genome assemblies were generated from Illumina sequencing data and evaluated using QUAST. Assembly statistics include total genome size, GC content, number of contigs, largest contig size, N50, and mean sequencing coverage. N50 represents the contig length at which 50% of the assembled genome is contained within contigs of equal or greater length. Mean coverage represents the average sequencing depth across the assembled genome based on read mapping statistics. These metrics were used to assess assembly quality and suitability for downstream genome mining and biosynthetic gene cluster analyses.

**Table 4:** Bioactivity profile of *Bacillus* cell-free supernatants.

*Bacillus* Strain ID	Indicator Organism	Agar-Well Diffusion Zone (mm, Mean ± SEM)	Drop-Collapse Assay Score (mm)	Arnow’s Test (A510 and Color Result ^1^)	Proteinase K Sensitivity ^2^	Thermal Stability ^3^
AD12	*E. coli*	20.3 ± 0.5	6 (6)	0.67 ± 0.02 (positive)	Resistant	Stable
	*S. aureus*	24.3 ± 1.2				
AD13	*E. coli*	19.2 ± 0.4	6 (6)	0.58 ± 0.03 (positive)	Resistant	Stable
	*S. aureus*	20.2 ± 0.7				
	*P. aeruginosa*	22.2 ± 0.8				
	*K. pneumoniae*	21.4 ± 0.4				
	*A. baumannii*	18.5 ± 0.3				
AD14	*E. coli*	17 ± 0.7	10 (9–10)	0.12 ± 0.01 (negative)	Sensitive	Stable
	*S. aureus*	16.4 ± 0.9				
	*P. aeruginosa*	19 ± 0.4				
AD15	*E. coli*	20 ± 0.9	11 (11)	0.15 ± 0.02 (negative)	Sensitive	Stable
	*S. aureus*	21.2 ± 0.5				
	*P. aeruginosa*	20 ± 0.3				
AD16	*E. coli*	24.1 ± 0.3	12 (10–12)	0.18 ± 0.01 (negative)	Sensitive	Stable
	*S. aureus*	23.2 ± 0.2				
	*P. aeruginosa*	22.2 ± 0.9				

This table represents the spectrum of antimicrobial activity of each CFS. Agar-well diffusion assays are represented by zones of inhibition (in mm) and are expressed as mean ± SEM. Drop-collapse assay results are represented by the median drop size of the CFS (in mm) compared to the drop size of the negative control (6 mm). Arnow’s test results represent absorbance (A5_10_) represented by mean ± SEM. Each experiment was repeated in triplicate. ^1^ Arnow’s test results based on color change (orange–red color indicates a positive reaction, yellow indicates a negative reaction), ^2^ sensitive = activity lost after treatment, resistant = activity retained after treatment, ^3^ stable activity retained after heating, sensitive = activity lost after heating.

**Table 5:** Antimicrobial activity and LC–MS/MS analysis of active fractions.

Fraction	Tentative Metabolite Annotation	Indicator Organism	MIC (µg/mL)	MBC (µg/mL)	RT (min)	Representative *m*/*z*	Major Fragments
F1	Tentatively assigned siderophore-associated metabolites	*E. coli*	32	64	6.42	883.3	721.2, 565.1, 447.1
F1	Tentatively assigned siderophore-associated metabolites	*K. pneumoniae*	32	64	6.42	883.3	721.2, 565.1, 447.1
F2	Unassigned molecular feature	*A. baumannii*	64	128	9.18	912.4	750.3, 594.2, 476.1
F2	Unassigned molecular feature	*P. aeruginosa*	64	128	9.18	912.4	750.3, 594.2, 476.1

Representative base peak chromatograms showed distinct metabolite profiles for fractions F1 and F2. Fraction F1 contained a dominant ion at *m*/*z* 883.3 with a retention time of 6.42 min and fragment ions at *m*/*z* 721.2, 565.1, and 447.1. Based on accurate mass and MS/MS fragmentation patterns, this molecular feature was tentatively assigned to a siderophore-associated metabolite class. Fraction F2 contained a dominant ion at *m*/*z* 912.4 with a retention time of 9.18 min and fragment ions at *m*/*z* 750.3, 594.2, and 476.1. This molecular feature could not be confidently matched to a known metabolite in the databases examined and therefore remained unassigned. MIC: Minimum inhibitory concentration; MBC: Minimum bactericidal concentration; RT: Retention time.

**Table 6:** Indicator bacteria used in this study and their resistance profiles.

Indicator Bacteria	Resistance Profile	Institute/Company
*Staphylococcus aureus* Y27	MET, VA	Medical Laboratory Sciences, Faculty of Allied Health Sciences, Kuwait University
*Klebsiella pneumoniae* ADA62	AMK, AMC, AMP, CTX, FOX, CAZ, CRO, CXM, CEF, CIP, GM, PIP	Clinical Microbiology Laboratory, Al-Amiri Hospital, Kuwait
*Escherichia coli* ADA165	AMK, AMC, AMP, CTX, CAZ, CRO, CXM, CEF, CIP, PIP	Clinical Microbiology Laboratory, Al-Amiri Hospital, Kuwait
*Acinetobacter baumannii* I-160	AMK, AMC, AMP, CTX, FOX, CAZ, CRO, CXM, CEF, CIP, GM, IPM, MEM, PIP	Clinical Microbiology Laboratory, Al-Amiri Hospital, Kuwait
*Pseudomonas aeruginosa* Y40	AMK, AMC, AMP, CTX, CAZ, CRO, CXM, CEF, CIP, GM, IPM, MEM, PIP	Medical Laboratory Sciences, Faculty of Allied Health Sciences, Kuwait University

Abbreviations correspond to resistance to VA; vancomycin, MET; methicillin, AMK; amikacin, AMC; Amoxicillin/clavulanic acid, AMP; ampicillin, CTX; cefotaxime, FOX; cefoxitin, CAZ; ceftazidime, CRO; ceftriaxone, CXM; cefuroxime, CEF; cephalothin, CIP; ciprofloxacin, GM; gentamicin, IPM; imipenem, MEM; meropenem, PIP; piperacillin.

## Data Availability

The draft genome assemblies generated in this study have been deposited in the NCBI GenBank database. The accession numbers are JBWXEP000000000 for AD12, JBSQAP000000000 for AD13, JBSQAQ000000000 for AD14, JBSQAR000000000 for AD15, and JBSQAS000000000 for AD16. The genome sequence data are publicly available through the National Center for Biotechnology Information (NCBI).

## Supplementary Materials

The following supporting information can be downloaded at: https://www.mdpi.com/article/10.3390/antibiotics15090863/s1, Table S1: RAST features of AD12–16 *Bacillus* strains, Table S2: Antimicrobial resistance determinants in strains AD12–AD16, Table S3: Antimicrobial resistance determinants and mobile genetic elements of AD12–16 *Bacillus* strains.

## Abbreviations

The following abbreviations are used in this manuscript:

AMR	Antimicrobial resistance
ANI	Average Nucleotide Identity
BGC	Biosynthetic gene cluster
CFS	Cell-free supernatant
DNA	Deoxyribonucleic acid
LC–MS/MS	Liquid chromatography–tandem mass spectrometry
MBC	Minimum bactericidal concentration
MIC	Minimum inhibitory concentration
mm	millimeter
NMR	Nuclear magnetic resonance
NRPS	Non-ribosomal peptide synthetase
PCR	Polymerase chain reactin
rpm	Rotations per minute
SEM	Standard error of mean
SM	Secondary metabolites
SPE	Solid-phase extraction
SPSS	Statistical Packages for Social Sciences
T3PKS	Type 3 polyketide synthase
TSB	Tryptone Soy broth
WGS	Whole-Genome Sequence
WHO	World Health Organization
XDR	Extensively drug-resistant
