# Supplementary material for: Genomic and Phenotypic Characterization of Sandstorm-Derived Airborne Bacillus Strains Reveals Diverse Biosynthetic Potential
Source: Antibiotics (Basel). 2026 Sep 4;15(9):863. doi: 10.3390/antibiotics15090863 (PMC13603614; doi:10.3390/antibiotics15090863)
Supplement: Supplementary file 1 [file antibiotics-15-00863-s001.zip › antibiotics-4460897-supplementary.pdf]

Table S1: RAST features of AD12-16 *Bacillus* strains

RAST features	AD12	AD13	AD14	AD15	AD16
Genes assigned to subsystems	1360	1405	1520	1585	1575
Virulence, disease and defense	58	63	71	75	73
Resistance to antibiotics	32	35	41	43	42
Stress response	75	80	88	93	91
Iron acquisition/metabolism	35	40	42	44	43
Secondary metabolism	26	31	36	39	38

Table S2: Antimicrobial resistance determinants in strains AD12–AD16

Strain	ARG determinant	Identity (%)	Coverage (%)	Accession	Antimicrobial class	Resistance mechanism
AD12	*bmrC*	94.8	98.6	WP_498831821	Multidrug	Antibiotic efflux
	bla (class D β-lactamase)	91.7	96.4	WP_129547731.1	β-lactam	Antibiotic inactivation
AD13	*bmrC*	95.5	99.0	WP_503373130.1	Multidrug	Antibiotic efflux
	bla (class D β-lactamase)	93.1	97.5	WP_103132055.1	β-lactam	Antibiotic inactivation
	Isa	97.4	99.0	WP_024423310.1	Multidrug	Antibiotic efflux
	bcrA	98.6	98.8	WP_198456513.1	Bacitracin	Cell envelope modification
AD14	*bmrA*	98.1	100	WP_410617837.1	Multidrug	Antibiotic efflux
	bla (Class A β-lactamase)	96.8	99.3	WP_425671150.1	β-lactam	Antibiotic inactivation
	bla (Class D β-lactamase)	98.8	99.5	WP_088325233.1	β-lactam	Antibiotic inactivation
	bcrC	97.4	98.5	WP_015714854.1	Bacitracin	Cell envelope modification
AD15	*bmrR*	98.4	99.4	WP_015251750.1	Multidrug	Antibiotic efflux
	bla (Class A β-lactamase)	96.8	99.0	WP_032725891.1	β-lactam	Antibiotic inactivation
	bla (Class D β-lactamase)	97.4	98.7	WP_003246349.1	β-lactam	Antibiotic inactivation
	lmrB	98.3	98.4	WP_003234772.1	Multidrug	Antibiotic efflux
	bcrC	97.7	98.9	WP_085187663.1	Bacitracin	Cell envelope modification
AD16	*bmrR*	95.8	100	WP_015251750.1	Multidrug	Antibiotic efflux
	bmrD	97.9	99.0	WP_103804001.1	Multidrug	Antibiotic efflux
	bmrC	98.8	97.4	WP_003245157.1	Multidrug	Antibiotic efflux
	bla (Class A β-lactamase)	96.5	99.0	WP_032725891.1	β-lactam	Antibiotic inactivation
	bla (Class D β-lactamase)	98.3	100	WP_003246349.1	β-lactam	Antibiotic inactivation
	ykkC	98.8	100	WP_003232583.1	Multidrug	Antibiotic efflux
	vmlR	95.6	98.3	WP_106610932.1	Lincomycin	Antibiotic target protection

Table S3. Antimicrobial resistance determinants and mobile genetic elements of AD12-16 *Bacillus* strains

Genomic feature	AD12	AD13	AD14	AD15	AD16
**AMR determinants (CARD/ResFinder)**	2	4	3	4	5
Multidrug efflux-associated determinants	+	+	+	+	+
β-lactam resistance-associated determinants	+	+	+	+	+
Tetracycline resistance-associated determinants	-	-	-	-	+
Macrolide/lincosamide-associated determinants	-	+	-	+	+
Bacitracin-associated resistance determinants	-	+	+	+	+
**PlasmidFinder**					
Known plasmid replicon detected	-	+	-	+	+
Number of plasmid replicons	0	1	0	1	1
Plasmid replicon type	-	Rep-type A	-	Rep-type B	Rep-type B
**Mobile genetic elements**					
Insertion sequence-associated elements	4	5	4	6	5
Transposon-associated elements	1	2	2	2	2
Total putative MGEs	5	7	6	8	7
AMR determinant on same contig as MGE	-	+	-	+	+

AMR, antimicrobial resistance; CARD, Comprehensive Antibiotic Resistance Database; MGE, mobile genetic element; +, detected; −, not detected.
